# Advances, applications, and prospects in aquatic botany

**DOI:** 10.1002/aps3.11488

**Published:** 2022-08-09

**Authors:** Julia A. Cherry, Gregory J. Pec

**Affiliations:** ^1^ Department of Biological Sciences University of Alabama Tuscaloosa Alabama 35487 USA; ^2^ New College University of Alabama Tuscaloosa Alabama 35487 USA; ^3^ Department of Biology University of Nebraska at Kearney Kearney Nebraska 68849 USA

Aquatic ecosystems, both freshwater and marine, compose a rich diversity of habitats that are increasingly recognized as vital to sustaining ecological stability and supporting human economic activity (Hofstra et al., 2020). Within these critical ecosystems, macrophytes, both native and invasive, represent less than 1% of the total vascular plant diversity, but they play vital roles in aquatic ecosystem structure (i.e., habitat heterogeneity and biodiversity) and function (i.e., nutrient and water cycling) (Havel et al., 2015; Geist and Hawkins, 2016; Hofstra et al., 2020). Despite their ecological importance, aquatic plants are among the most threatened groups of species worldwide due to land‐use change, modified water regimes, and effects of climate warming (Chambers et al., 2008; Hilt et al., 2017). These threats can have profound effects on aquatic plant diversity, productivity, and function, and, in turn, how we manage, protect, and conserve these systems.

In recent decades, technological advances in analytical and survey methodologies have more readily been applied to aquatic plant research and provide an important means to enhance understanding of aquatic plant distribution and survivorship as well as biotic interactions with invasive species and abiotic interactions with the environment (O'Hare et al., 2018). For example, cost reductions and minimization of repeat sampling of sensitive species and/or habitat have allowed for the broader use of stable isotope analysis in aquatic systems (e.g., Glibert et al., 2019), while continued developments in ecological modeling and computational biology have improved our understanding of complex interactions with aquatic plant species (e.g., Wood et al., 2014; Boothroyd et al., 2015; Verschoren et al., 2016). In this special issue of *Applications in Plant Sciences*, “Advances, applications, and prospects in aquatic botany,” we present four papers that explore current methods and challenges in two key areas of aquatic plant research: (i) biodiversity and conservation and (ii) aquatic invasive species management.

## Biodiversity and conservation

Our first paper in this issue (Tyrrell et al., 2022) presents a novel trait‐based approach to monitoring macrophyte systems. Historically, compositional‐ and diversity‐based surveys were challenging due to the lack of taxonomic resolution and overall sampling effort. Methodological improvements have increased our ability to identify, map, and relate diversity metrics to quality indices of the aquatic environment (Visser et al., 2015; Spears et al., 2016). However, these metrics are often local or regional in focus due to the strong influence of the physico‐chemical environment as well as less generalizable when using simplistic taxonomic‐based approaches (McGill et al., 2006; O'Hare et al., 2018). Here, Tyrrell et al. (2022) explore the possibility of adapting macrophyte‐based metrics (i.e., growth‐form trophic affinity derived from species trophic affinity) from one geographic region (Europe) to evaluate trophic water conditions in another geographic region (Canada). They demonstrate that adopting aquatic plant growth form instead of taxonomic identity provides an improved relationship with actual trophic water conditions. They suggest that this mechanistic index provides an alternative bioassessment application tool and offers the ability for inter‐regional or inter‐continental comparisons.

Our second paper in this section (Lane, 2022) looks more closely at plant community composition in estuaries, in particular tidal freshwater marshes (TFMs) in the upper reaches of an estuary. These habitats are vital for carbon storage, nutrient cycling, and habitat for migratory salmon and seabirds. However, due to the loss of TFMs from human developments, there is an increased need to better understand and conserve these habitats (Mueller et al., 2016; Chalifour et al., 2019). Specifically, studies on aquatic plant recruitment from seed in TFMs represent a significant knowledge gap. Lane (2022) highlights the importance of germination ecology in TFMs and reports on how marsh organs can be used to study germination processes in tidal conditions. The author looks at the effects of artificial and natural chilling as well as the presence and/or absence of near‐neighbor aquatic transplants on germination of five TFM species based on their habitat prevalence and commercial availability. Lane (2022) illustrates an easy and cost‐effective field‐based approach that can be applied to different locations and environmental conditions, and provides insight into identifying species‐specific seed recruitment niches for restoration or conservation applications.

## Aquatic invasive species management

Generally, the pace of current biological invasions exceeds that of previous events that occurred over geological time scales (Ricciardi, 2007). Invasive species in aquatic ecosystems have a variety of impacts on biodiversity and ecosystem function. Although some aquatic invasive species can have little to no effect on the environment (e.g., Havel et al., 2005), many have significant negative effects on other species and the environment generally (e.g., Bunn et al., 1998). As a result, aquatic invasive species pose challenges to the restoration or conservation of many aquatic habitats. Our first paper in this section (Van De Verg and Smith, 2022) outlines a novel, field‐based methodology using a common biodegradable chemical for mitigating an invasive macroalga. Here, Van De Verg and Smith (2022) administer differing concentrations of hydrogen peroxide into individual basal attachments of the invasive seaweed *Avrainvillea lacerata* within an impacted reef flat. They found a significant reduction in relative electron transport rate maxima (a measure of photosynthesis) following injection of hydrogen peroxide, and the authors discuss the possible utility of this method at larger scales.

Along with the impact aquatic invasive species have on species composition and abundance, they are also known to restructure food webs, particularly in freshwater ecosystems (see Havel et al., 2015 and references therein). However, little is known about food web impacts of aquatic invasive plants on higher trophic level changes. Our remaining contribution to this issue, by Wigginton et al. (2022), highlights the use of stable isotopes and Bayesian mixed modeling to examine the role of an invasive aquatic plant on resource use of song sparrows. They demonstrate that song sparrows showed reliance on the seeds of the invasive plant *Lepidium latifolium* as well as seasonal differences in resource use. The use of advanced tools (i.e., stable isotope analysis and Bayesian mixed modeling) has important implications for invasive plant control and management, as attempts to control invasive plants could have negative or unintended consequences on other species that rely on them for trophic support.

Overall, these papers present work at the cutting edge of aquatic botanical research. Our understanding of aquatic plant biology and ecology has never been greater, particularly with the increased range of new techniques and approaches becoming more readily available. Historic “wait‐and‐see” approaches to biodiversity, invasive species control, and conservation are not a viable option. More rapid, cost‐effective, and robust methods and approaches—as highlighted in this special issue—are critical for the preservation of current aquatic ecosystems and the services they provide. We hope that you find these articles both informative and inspirational in this dynamic and ever‐changing field of aquatic botany.

## AUTHOR CONTRIBUTIONS

G.J.P. prepared the first draft of the manuscript. J.A.C. and G.J.P. edited the subsequent drafts. Both authors approved the final version of the manuscript.
